# Lightwave Circuits in Lithium Niobate through Hybrid Waveguides with Silicon Photonics

**DOI:** 10.1038/srep22301

**Published:** 2016-03-01

**Authors:** Peter O. Weigel, Marc Savanier, Christopher T. DeRose, Andrew T. Pomerene, Andrew L. Starbuck, Anthony L. Lentine, Vincent Stenger, Shayan Mookherjea

**Affiliations:** 1 Department of Electrical & Computer Engineering, University of California, San Diego, La Jolla, California 92093, USA; 2 Sandia National Laboratory, Applied Microphotonic Systems, Albuquerque, New Mexico 87185, USA; 3 SRICO Inc., 2724 Sawbury Blvd Columbus, OH 43235-4579, USA

## Abstract

We demonstrate a photonic waveguide technology based on a two-material core, in which light is controllably and repeatedly transferred back and forth between sub-micron thickness crystalline layers of Si and LN bonded to one another, where the former is patterned and the latter is not. In this way, the foundry-based wafer-scale fabrication technology for silicon photonics can be leveraged to form lithium-niobate based integrated optical devices. Using two different guided modes and an adiabatic mode transition between them, we demonstrate a set of building blocks such as waveguides, bends, and couplers which can be used to route light underneath an unpatterned slab of LN, as well as outside the LN-bonded region, thus enabling complex and compact lightwave circuits in LN alongside Si photonics with fabrication ease and low cost.

Lithium Niobate (LN) was once considered the most promising of materials for integrated optics^1^, but despite a rich set of properties, the technology of LN integrated optics has not evolved as much as integrated optics in III-V semiconductors and silicon (Si) photonics^2^,^3^. Although high performance stand-alone LN devices have been shown^4^,^5^ and a technique of ion-sliced thin-film LN has been developed^6^,^7^,^8^, the technology of LN integrated optics has continued to rely on traditional waveguide fabrication techniques based on ion-exchange^9^, diffusion and serial writing^10^ or mechanical sawing^11^,^12^, all of which are very different from the modern lithographic techniques and foundry processing available in Si or III-V photonics.

To address this problem, we develop an approach to developing hybrid waveguides in LN based on a two-material core cross-section, consisting of few-hundred nanometer single-crystal thin films of Si and LN bonded face-to-face. We used manufacturer-provided Si-on-insulator and LN-on-insulator wafers which have a 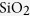 cladding layer which is a few microns thick, and a handle material (Si and LN, respectively) for the substrate which is several hundred microns thick. We first pattern all features required for waveguiding in the Si wafer using deep ultraviolet (DUV) lithography (see Methods section). This allows much finer features to be patterned than is possible in LN (especially in comparison to conventional thin film LN waveguides^13^). Since LN is not a CMOS-compatible material and is not processed in CMOS foundries, we developed designs which allow all the waveguiding circuitry to be defined in Si, requiring only a single back-end process step of bonding LN to form the functional chip. DUV lithographic fabrication of Si photonic features is more precise, scalable and manufacturable than the traditional waveguide-fabrication techniques in LN, and the LN layer can be left as an unpatterned slab that is then bonded to the patterned Si^14^. By not patterning, etching or sawing LN, its material properties are kept pristine^15^.

Figure 1 shows images of a bonded chip whose dimensions are that of a typical field size of a stepper projection DUV lithography system (few centimeters squared). Upon bonding, a hybrid waveguide is formed, in which the geometric dimensions of the Si features and LN film dictate how the optical mode is distributed between the Si and LN layers. Techniques have been developed in the past decade for bonding LN of various orientations to Si wafers without incurring thermal stress mismatches^16^,^17^,^18^,^19^,^20^,^21^. Bonding achieves much higher quality and better optical properties than growth of LN on Si using sol-gel processes^22^ or chemical vapor deposition^23^. We directly bonded the LN chip to the Si chip i.e., without any intervening polymeric ‘glue’ layer as used in some approaches^13^,^24^, thus permitting the maximum control over the mode distribution.

As will be discussed below, light propagates mainly in the silicon layer in certain sections of the layout, and primarily in the LN layer where desired, and makes transitions between the two layers at several locations while remaining in the transverse-electric (TE) polarized single-mode regime, which is highly desirable for Si photonics. Thus, we can also design devices that are outside the Si-LN bonded region and behave entirely like conventional Si photonics. In this way, the technology for Si photonics can be leveraged to enhance LN integrated optics, and vice versa.

The main advantages of this approach are: (a) the requirements on LN fabrication are reduced to a minimum, i.e., a single bonding step to the manufacturer-supplied wafer after all silicon patterning has been completed (etching LN is possible^25^,^26^ but is technologically less mature than etching Si, which is available in every foundry process); and (b) both the cross-sectional mode area and the length of components are greatly reduced, compared to traditional LN ion-exchanged or diffused waveguides, allowing complex circuits to be realized in a small area. The tradeoffs are that: (a) the bonding step should be performed at a low temperature since Si and LN have different coefficients of thermal expansion, and (b) the resulting hybrid modes have a higher refractive index dispersion compared to low-index contrast (e.g., doped-glass) integrated optics, and thus, components will be optimized for a desired vacuum wavelength range, e.g., the telecommunications band around 1550 nm.

## Hybrid Modes and Mode-Transition Tapers

The dimensions of the Si features and LN film determine what fraction of the optical mode resides in Si and in LN. Two distinct waveguide cross-sections, labeled ‘A’ and ‘B’, are selected, as shown in Fig. 2a, and used in the microchip layout. These cross-sections vary only in the width of the Si structures, which allows for convenient fabrication in a single step of lithography. The numerical values of the widths and heights chosen here are suitable for wavelengths between 1530 nm and 1565 nm (i.e., the telecommunications C-band).

**Figure 1:**
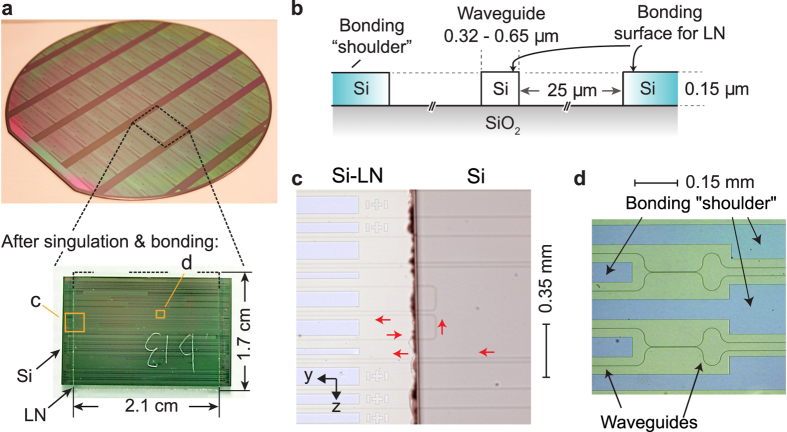
Hybrid LN-Si photonic circuits. (**a**) Silicon photonic components were fabricated using deep ultraviolet (DUV) lithography. Singulated dies (size: 25 mm × 16 mm) were bonded to diced pieces (size: 21 mm × 17 mm) of an unpatterned Lithium Niobate (LN)-on-insulator wafer. This particular LN chip was labeled ‘b13′ by scratch marks on the LN substrate (0.5 mm thick, opposite to the bonded surface). (**b**) Schematic of the cross-section showing how waveguides and bonding “shoulders,” which are at the same height as the waveguides, are conveniently formed in one lithographic etch step on the Si wafer. (**c**) Optical microscope image showing waveguides transitioning between the portion of the hybrid chip which is not covered by LN (i.e., conventional 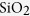-clad Si photonic waveguides), and that which is bonded to LN (and uses hybrid waveguides). Arrows colored red indicate the back-and-forth direction of light propagation in certain representative sections. (**d**) Optical components can be defined in the 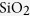-clad Si section, or in the LN-Si bonded section.

**Figure 2:**
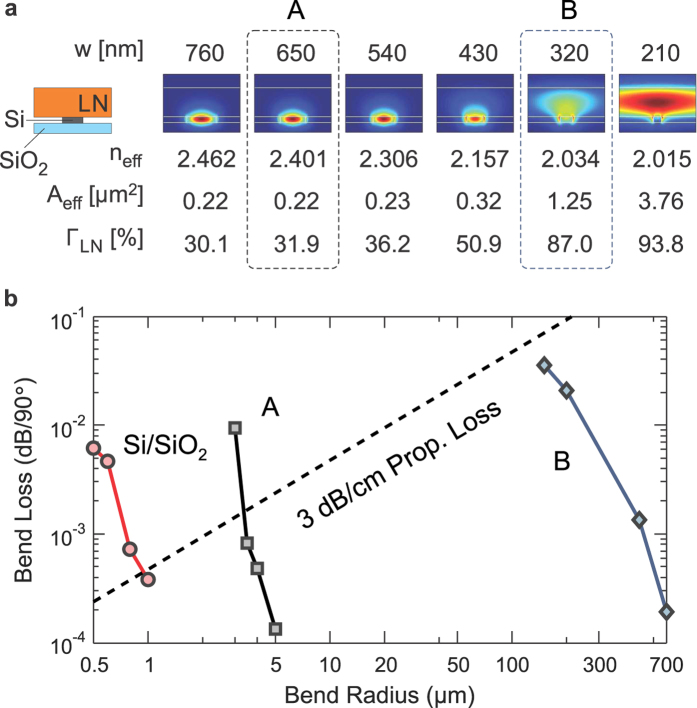
Waveguide modes. (**a**) Calculated hybrid optical mode profiles for different Si rib widths. The panels show the magnitude of the electric field in the TE polarization, with the E-field vector oriented along the crystal axis. As the Si rib width 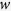 decreases, the modal effective index (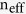) decreases, the effective area (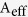) increases, and the fraction of light in LN (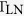) also increases. The dotted boxes indicate the two cross-sectional designs (‘A’ and ‘B’) used in the chip. (**b**) The calculated bending loss of the ‘A’ cross-sectional mode is much lower than that of the ‘B’ mode, and is not too different from the waveguides used in silicon photonics (labeled as ‘Si/SiO_2_’). Therefore, ‘A’ is used for bends and compact routing, and ‘B’ is used in straight waveguide sections when most of the light should “see” LN. The dashed line labeled ‘3 dB/cm Prop. Loss’ represents the propagation loss at all bend radii for a propagation loss of 3 dB/cm to give an idea of practical minimum losses for bent waveguides.

Cross-section A consists of 650 nm wide and 150 nm tall Si features, bonded to LN. The lowest-order mode of the waveguide is quasi-TE-polarized and is similar to the quasi-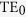 mode in conventional silicon photonics (see Fig. 2a). Wider waveguides are close to becoming multi-moded, which is undesirable. A waveguide using cross-section A can transition from an 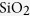-clad silicon photonic section to the bonded Si-LN region, as shown in Fig. 1c, with less than −0.3 dB loss calculated by numerical simulation (Lumerical software package). We have not experimentally confirmed the mode-transition loss at this time. As shown by Fig. 2b, waveguides using cross-section A can support compact bends with radii of 3–5 μm without incurring bending losses higher than that of a standard rib waveguide (650 nm × 220 nm surrounded by SiO_2_, labeled ‘Si/SiO_2_’ in Fig. 2b) with a radius of 0.5–1 μm.

Cross-section B consists of 320 nm wide Si features at the same height as cross-section A. With an air upper cladding, this waveguide is unable to support a waveguide mode by itself, and thus requires the bonded LN layer to support a well-defined mode at C-band wavelengths. Cross-section B cannot be used for compact low-loss bends (see Fig. 2b). Instead, it is used when we want the light to interact with the crystal properties of LN; otherwise, cross-section A is used to route light on the hybrid chip. Therefore, it is desirable to maximize the fraction of light in LN for the mode in cross-section B. The confinement factor is defined as follows: 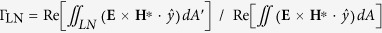, where 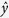 is the direction of light propagation and the range of integration in the integral in the numerator is restricted to the LN region. In waveguides using cross-section A, 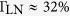 whereas in waveguides using cross-section B, 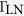 ≈ 87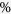. The transition between the two cross-sections is discussed below.

A modified version of cross-section A, in which 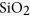 replaces LN as the upper cladding, is used outside the LN bonded region to demonstrate all-Si components made on the same platform and at the same device level. Another cross-section with a reduced Si width of 180 nm is used for couplers at the edge of the Si chip, as is commonly used in Si photonics^27^, and can also be fabricated in the same step.

All these waveguide shapes are simple rectangles without relying on slots, tilted sidewalls or other difficult-to-fabricate shapes. There is no patterning in the LN layer which eliminates many of the challenges faced in the past^13^. Conceptually, the waveguide structure is similar to that of the strip-loaded waveguide film studied in the 1970’s^28^, but scaled to the deep sub-micron regime. The modal area of cross-section A (0.22 μm^2^) is, in fact, smaller, and that of cross-section B (1.25 μm^2^) is only slightly larger, than those of the smallest-area waveguides reported in LN, fabricated by the ion-milling technique^29^.

As shown in Fig. 1d, large rectangular areas were defined in the Si device layer at distances of about 30 μm from the waveguide edge. These “bonding pads” between the Si and LN chips are far from the waveguide core and serve no optical purpose, but instead provide a large surface area for bonding. They are precisely at the same height as the Si rib features, since they are formed in the same lithographic step. They are similar to “dummy fill” features inserted to assist in chemical-mechanical polishing, but without a fragmented pattern. The etched trenches between the Si ribs and the bonding pads not only provide optical confinement but may assist in bonding by defining convenient outgassing channels, and for local stress relaxation.

**Figure 3:**
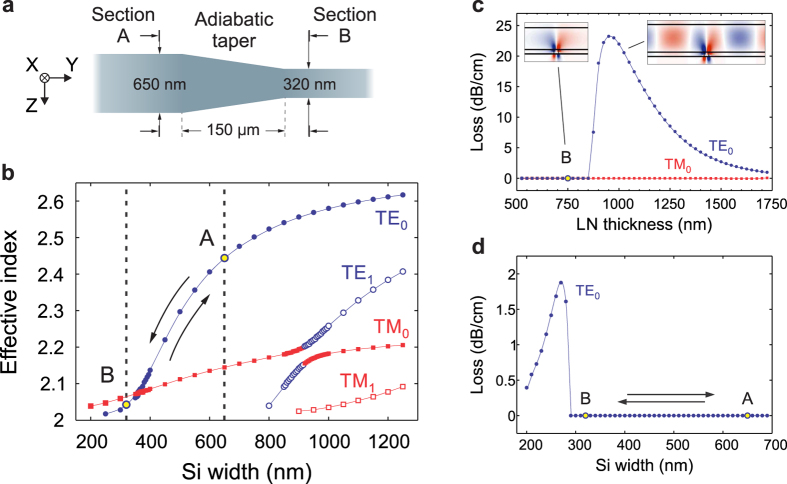
Adiabatic mode transition. (**a**) Gradual linear reduction of the Si rib width to transition between cross-sections A and B. (**b**) Numerical calculations of the modal effective index versus waveguide width, with yellow circles indicating the initial and final points of the taper. The TE-polarized mode does not hybridize with, or convert into, the TM-polarized mode in the taper. (**c**) Plot of simulated propagation losses for cross-section B with a varying LN film thickness. (Inset: E_x_ component of the fundamental TE_0_ mode, (left) bound for cross-section B, i.e., 750 nm LN; and (right) leaky for 1000 nm thick LN.) The losses decrease as the mode approaches a TE slab mode. (**d**) Plot of simulated propagation losses for our hybrid geometry (150 nm tall Si, 750 nm thick LN) with a varying Si width. Similar to (**c**), losses increase suddenly when the x-component of the E-field is no longer confined, and then decrease as the mode approaches a TE slab mode.

Along the direction of light propagation, adiabatic tapered transitions were defined in the Si layer (see Fig. 3a) as a linear change in the Si rib width over a distance of 150 μm. The quasi-TE-polarized mode 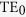 has the highest effective refractive index in cross-section A (and is used extensively in conventional Si photonic waveguides, which are wider than they are tall^30^). In cross-section B, it is actually the quasi-TM-polarized mode 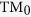 which has the higher refractive index. This is due to both the anisotropic properties of LN (LN has a lower material index along the z-axis) and the low index lateral air cladding on either side of the Si. However, the taper-induced coupling between these modes is zero to first order because the electric field of the former is a symmetric mode, and that of the latter is an anti-symmetric mode^31^. Thus, we are able to draw the waveguide down to a narrow width that pushes a very large fraction of the light into LN while maintaining its state of polarization all the way from the feeder waveguides. For the Si rib widths used in this design, the higher-order quasi-TE polarized mode (labeled 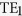 in Fig. 3b) is not guided; however, by using wider Si rib widths, tapers can couple between this anti-symmetric mode and the 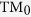 mode, which may be useful for polarization rotators etc. Thus, compared to other hybrid Si-LN structures^20^,^26^,^32^, our design achieves the highest TE-polarized (lowest-order) mode-fraction in LN while also providing a pathway for integration to Si photonics by first coupling light into cross-section A (which is similar to a traditional Si photonics cross-section), and then transitioning into cross-section B adiabatically. Attempting to couple from an external input to the quasi-TE mode in cross-section B would render the device very susceptible to roughness and bending losses, because it is not the fundamental mode. Widening the Si waveguide effectively pulls the light back into Si again, and, because it remains in the TE-polarization, allows for the benefits that have been realized by Si-only waveguides, such as tight bends and compact directional couplers, among others.

Another important consideration is that the propagation loss of a hybrid mode can increase significantly due to lack of transverse confinement, if the LN thickness falls within a range of values, depending upon the Si rib dimensions, at which one of the vectorial field components becomes slab-like (in the LN region). This effect, studied and measured in standard rib waveguides^33^, has recently been highlighted for quasi-TE modes in x-cut LN proton exchanged channel waveguides^34^. Similarly, in our configuration, it originates from the TE 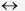 TM mode coupling at the Si strip boundaries. Quasi-TE mode leakage then occurs when the modal effective index becomes smaller than that of the TM slab mode in LN, i.e., when the minor TM-polarized field is no longer a bound mode and propagates in the LN planar waveguide. Due to the LN birefringence, this condition cannot be met for the quasi-TM modes which is therefore immune to leakage. This is shown in Fig. 3c: for LN thicknesses between 850 nm and 1750 nm (not used in our present design), the propagation loss would be significantly higher than at other thicknesses. A similar behavior is observed when the width of the Si region is reduced and the LN thickness is fixed, as in Fig. 3d.

## Waveguides, Directional Couplers and Photonic Circuits

**Figure 4:**
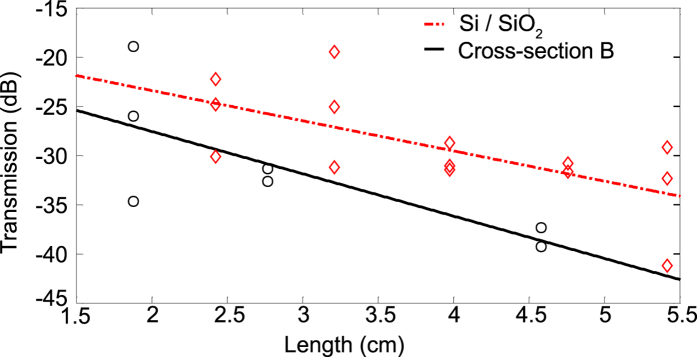
Waveguide characterization. Transmission versus length of waveguides and bends (paper-clip structures) at the wavelength of 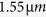 for mode cross-section ‘B’ in the straight sections and cross-section ‘A’ in the semi-circular bends (labeled ‘Cross-section B’). The propagation losses of test structures consisting of 650 nm wide Si waveguides with 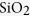 cladding are also shown (labeled ‘Si/SiO_2_’). The numerical fit is shown by the lines.

Photonic circuits can be assembled from the basic building blocks of waveguides and directional couplers. Hybrid waveguides of lengths between 1.8 cm and 4.6 cm were fabricated with the majority of the length consisting of waveguides using cross-section B. Cross-section A was used at the semi-circular bends, in order to fit the longer waveguides into a compact footprint using “paperclip” structures. The longest waveguide involves 10 transitions between cross-sections A and B. Since the edge facets were roughly diced and not polished, the fiber-to-chip insertion loss was high (about 9.5 dB without index-matching liquid) and non-uniform. As shown in Fig. 4, by measuring transmission versus length across several chips, we were able to build up an ensemble of measurements from which a propagation loss of 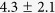 dB/cm was extracted at 1550 nm wavelength with only minor changes across the wavelength range 1530 nm to 1570 nm. At this time, we are unable to separate the bending loss from the straight waveguide propagation loss, but the former is expected to be very small based on the large bending radius of 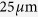. Numerical simulations reported in Fig. 2b suggest that the bending loss should drop to less than 0.001 dB per 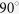 bend for bending radius greater than 3 μm using cross-section A.

By way of comparison, the propagation loss of the same Si waveguides with 3 μm 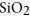 top-cladding (rather than LN) was measured to be 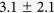 dB/cm, indicating that bonding and incorporation of LN as the top cladding did not significantly worsen the propagation characteristics. In fact, the propagation loss is similar to that measured in rib Si photonic waveguides^30^ despite the thinner Si layer in our structures.

The loss values in these hybrid Si-LN waveguides using cross-section B are significantly lower than other reported values, e.g., 16 dB/cm in etched thin-film LN waveguides^13^ and 6–10 dB/cm in thin-film LN waveguides (660 nm thickness, not too different from the 750 nm thickness used here) with oxidized titanium stripe^35^. Although the loss is still an order-of-magnitude higher than that of traditional in-diffused waveguides which have a much larger mode area, our circuits are also more than an order-of-magnitude more compact. Furthermore, we expect that with improved Si waveguide fabrication (e.g., roughness reduction), and filling of the air pockets on the lateral sides of the Si rib with a low-optical-loss gap-fill dielectric material, the overall propagation loss will decrease.

Directional couplers were defined by lithography in the Si layer, but act on the hybrid mode using cross-section A, with a gap of 0.4 μm between the waveguide edges. The typical length was only 150 μm, compared to a typical length of about 5 mm for directional couplers with diffused or ion-exchanged waveguides. Because the mode in cross-section A resides primarily in Si, there was no measurable crosstalk at milliwatt power levels, unlike the traditional waveguide LN devices which are susceptible to photorefractive artifacts^36^,^37^. However, long-term (>1 day) tests have not yet been performed since the chips are not fully packaged and we rely on manually-adjusted fiber coupling to the silicon waveguides.

**Figure 5:**
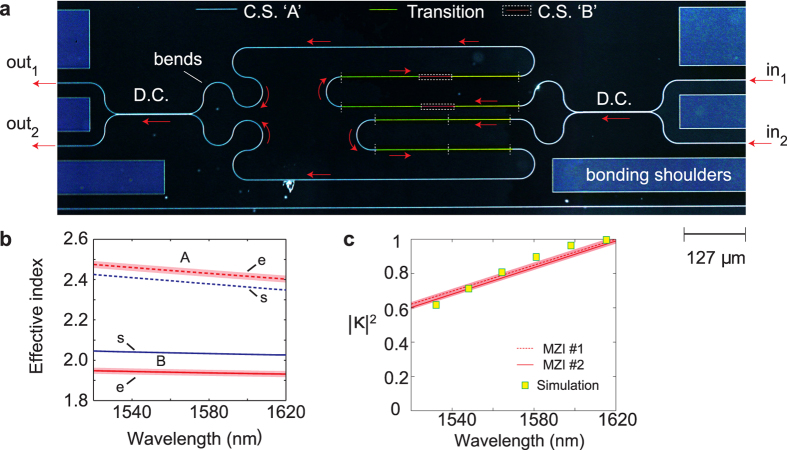
Hybrid Si-LN optical circuit. (**a**) Optical microscope image of an interferometeric hybrid Si-LN circuit which uses both waveguide cross-sections (C.S.) A and B, four adiabatic transitions (Transition) in each lightpath, two directional couplers (D.C.), and twenty 90-degree bends in each lightpath. The image is shown using a simulated dark-field colour map for clarity, and with added shading to highlight the different sections. (**b**) Extracted modal effective index versus wavelength for MZIs with path-length difference (PLD) regions comprised of hybrid waveguides with cross-sections A (red dashed lines) and B (red solid lines), labeled with “e.’’. Simulated values using an eigenmode solver software (A: blue dotted, and B: blue solid lines) are also shown (labeled with “s’’), assuming a nominal (design) Si rib waveguide width, and manufacturer-specified LN film thickness. Shaded regions indicate the standard errors provided by the fitting routine (see Supplementary Information). (**c**) Extracted coupling coefficient of the directional coupler versus wavelength for the MZIs reported here (dashed and solid lines). MZIs with PLD cross-sections A and B use the same type of directional coupler, hence the results are similar. The simulation results shown by the squares were calculated using the supermode equations and a numerical simulation of the eigenmodes. Shaded regions indicate the standard errors provided by the fitting routine (see Supplementary Information).

Optical circuits can be designed if two fundamental parameters are known: the optical (modal, i.e., “effective”) refractive index of a waveguide, which determines the rate of accumulation of optical phase with distance, and the directional coupling coefficient of two adjacent waveguides. Both these parameters are functions of the optical wavelength. To measure these parameters and compare with numerical calculations, we designed an interferometric test structure (see Fig. 5) which can also perform several functionalities useful in integrated optics such as spectral filtering, interleaving or multiplexing/de-multiplexing for wavelength-division multiplexing (WDM). Such a device does not use microring or standing-wave (e.g., Fabry-Perot) resonators, in which the intensity enhancement (due to the infinite impulse response nature of the transfer function) may cause photorefractive artifacts in LN. The circuit consists of several building blocks where the optical pathway is indicated by the red arrows and consists of four transitions, two directional couplers and twenty 90-degree bends, incurring a cumulative insertion loss of about -15 dB. (The circuit does not need to be so complicated to realize only a Mach-Zehnder interferometer, but showcases some of the capabilities of the platform.)

## Discussion

To experimentally show the large difference in the effective modal refractive index between the A and B cross-sections, we measured two distinct MZIs in which the path imbalance of one arm with respect to the other was formed using waveguides of cross-section A and B, of length 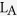 and 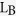, respectively. In each case, the free-spectral range (FSR) of the MZI was approximately 10 nm near the central wavelength of 1550 nm. The Supplementary Information shows the ‘Cross’ (in_1_ to out_2_ or in_2_ to out_1_) and ‘Thru’ (in_1_ to out_1_ or in_2_ to out_2_) transmissions measured at the two output ports for each input port (a total of four combinations for each MZI structure) while the laser wavelength was varied between 1520 nm and 1620 nm, and describes the data fitting procedure.

Figure 5b shows the wavelength variation of the effective refractive index of the A and B waveguides. The two branches (cross-section A and cross-section B) are sufficiently far apart that the results confirm a clear distinction between the A (Si-like) and B (LN-like) guided modes. The group velocity dispersion (GVD) coefficients of the two modes at 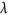 = 1550 nm are as follows: 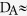 − 3100 ps/nm-km for cross-section A, and 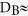 −4300 ps/nm-km for cross-section B, which are of the same order-of-magnitude as that of a typical silicon photonic waveguide whose modal effective area is similar to that of cross-section A, 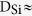 − 1500 ps/nm-km^38^.

Figure 5c shows the wavelength variation of the coupling coefficient of a directional coupler which was formed using waveguides of cross-section A, with a gap of 0.4 μm between the waveguide edges. As may be expected, the change in the magnitude of the coupling coefficient over the wavelength range of 1520 nm–1620 nm is similar to the behavior seen in all-Si waveguides^39^.

**Figure 6:**
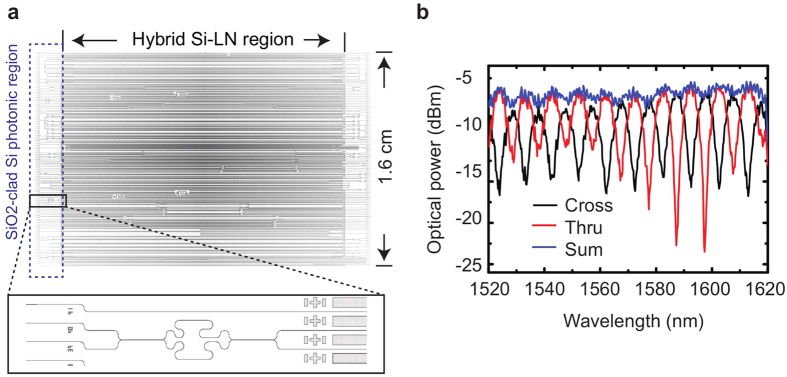
Si/SiO_2_ Mach-Zehnder interferometer (MZI). (**a**) An 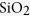-clad Si photonic MZI was fabricated on the same chip but outside the bonded LN region. The input-output waveguides pass under the LN bonded region and emerge on the other facet of the chip. (**b**) The measured ‘Thru’ (red) and ‘Cross’ (black) characteristics, and their sum (blue) follow the expected behavior for an MZI, and demonstrate that conventional Si photonic devices can be fabricated alongside the hybrid Si-LN ones (the y-axis is arbitrarily normalized).

On the same chip, outside the LN-bonded region, waveguides and devices may be designed as usual in Si photonics, i.e., with 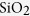 cladding. Because of the high refractive index of Si, light is tightly confined for waveguides of cross-section ‘A’ whether LN or 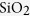 is used as the upper cladding. We designed and measured a Mach-Zehnder interferometer outside of the LN-bonded region, using waveguide with cross-section ‘A’ and with 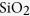 replacing LN as the upper cladding (and with 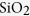 side- and lower-claddings in our fabrication process), as reported in Fig. 6. Although our test chip shown in Fig. 1 was designed for LN covering nearly all of the Si surface, it is equally possible to design “mixed” chips with smaller-sized LN pieces, which combine traditional Si photonic components with hybrid LN-Si photonic components on a monolithic platform.

We have restricted this fabrication process for reasons of cost and complexity to exclude dopants and electrodes as part of the Si chip, which could be used for electro-optic effects. Simulations show that gold electrodes can be positioned directly on the LN thin-film layer (surface opposite to the bonded interface to Si) at a lateral distance of only 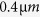 from the Si edge for cross-section A and 4 μm from the Si edge for cross-section B, for an estimated additional propagation loss of 0.1 dB/cm. Alternatively, electrodes can be fabricated on the LN layer after substrate removal, following the traditional approach^13^.

## Conclusion

These results show that, even though LN is not a CMOS-compatible material, foundry-fabrication technologies can play a very useful role in a new generation of LN integrated optics. While LN has always been a desirable material for its nonlinear and electro-optic properties, it has not been possible in the past to make compact and complex waveguide circuits as is possible nowadays in Si photonics using precise and highly-repeatable DUV lithography. We have shown a suite of hybrid building blocks from which optical circuits can be assembled alongside traditional Si photonics components. While improved wafer-scale bonding techniques are being developed by industry for commercial applications, here we have shown chip-scale direct bonding of chips that are a few centimeters squared (the size of a typical field size of a DUV stepper lithography system), with enough bond strength to permit dicing and simple packaging for test and measurement. A similar approach may also be applied to design and investigate optical circuits using other thin-film materials in place of LN, leveraging the advanced foundry fabrication capabilities of Si photonics as a waveguiding template for the hybrid modes without needing to pattern the thin-films.

## Methods

### Fabrication

The silicon features were patterned using DUV lithography on silicon-on-insulator wafers with a substrate oxide thickness of 3 μm at Sandia National Laboratories. A blanket etch was used for height reduction of the device layer from 250 nm to 150 nm followed by patterning to define waveguides and bonding pads simultaneously (i.e., at the same height). The thin-film LN on insulator wafers were procured from a commercial source (NanoLN Jinan Jingzheng Electronics Co. Ltd.), with a nominal top LN thickness of 750 nm, silicon oxide buffer of thickness 2 μm and LN handle of thickness 0.5 mm, diced into chips of size 2.1 cm × 1.7 cm. The materials properties of such thin-films have been documented elsewhere^15^.

### Bonding

There are several techniques that could be used for bonding Si to LN, most of which rely on managing the temperature budget of the process, in view of the different thermal coefficients of expansion of the two crystalline materials. In this work, we used a room-temperature bonding process without any intermediate layers, i.e., direct bonding, which relies on van der Waals or hydrogen bonding^16^,^40^. The cleaning process was as follows: 1) a solvent clean of the Si and LN chips in an ultrasonic bath; 2) a buffered oxide etch of the Si chip only for 4 seconds; 3) a piranha acid (2:1, H_2_SO_4_:H_2_O_2_) clean of the Si and LN chips for 5 minutes, followed by a two-stage water bath (10 sec. in first bath, 1 min. in second bath); 4) N_2_ blow dry both chips; 5) plasma surface activation (PSA) of both chips with O_2_ gas for 45 sec. at 200 W and 200 mT in a Technics PEIIB; 6) water bath for both chips immediately after the PSA, for at least 1 min.; 7) N_2_ blow dry both chips. After drying, the chips were aligned manually such that the z-axis of the (x-cut) LN was perpendicular to the long axis (y) of the optical waveguides. Since our direct bonding technique does not rely on high pressure, bonding was initiated at room temperature by gently pressing and sliding outwards from the center using plastic tweezers or the backside of a clean room swab. After bonding, approximately 825 nm of 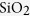 was sputtered at room temperature (Denton 635, 400 W, 5 mT; growth rate approximately 275 nanometers of 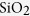 per hour).

We performed a bond strength analysis using an Imada force gauge and test stand to measure tensile “pull-apart” force^41^. The average bond strength measured over a few test bulk Si-LN chips was 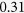 MPa for chips cleaned using an ultrasonic cleaner, and 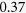 MPa for chips cleaned using a megasonic cleaner. While the bond strengths are not as high as commercially-bonded wafers, these results eliminate the possibility of the sputtered oxide being an ad-hoc “glue” holding the wafers together. These bond strengths were sufficient for handling and testing the chips, which have remained bonded for more than four months without any special storage techniques. However, additional processing to achieve stronger bonds will be required for commercial applications, which may involve proprietary recipes.

## Additional Information

**How to cite this article**: Weigel, P. O. *et al.* Lightwave Circuits in Lithium Niobate through Hybrid Waveguides with Silicon Photonics. *Sci. Rep.* **6**, 22301; doi: 10.1038/srep22301 (2016).

## Supplementary Material

Supplementary Information

## References

[b1] Lawrence M. Lithium niobate integrated optics. Rep. Prog. Phys. 56(3), 363 (1993).

[b2] Smit M., Van der Tol J. & Hill M. Moore’s law in photonics. Laser Photon. Rev. 6(1), 1 (2012).

[b3] Hochberg M. & Baehr-Jones T. Towards fabless silicon photonics. Nat. Photonics 4(8), 492–494 (2010).

[b4] Sohler W. *et al.* Integrated optical devices in lithium niobate. Opt. Photonics News 19(1), 24–31 (2008).

[b5] Chiba A. *et al.* 16-level quadrature amplitude modulation by monolithic quad-parallel Mach-Zehnder optical modulator. Electron. Lett. 46(3), 227–228 (2010).

[b6] Levy M. *et al.* Fabrication of single-crystal lithium niobate films by crystal ion slicing. Appl. Phys. Lett. 73, 2293 (1998).

[b7] Rabiei P. & Gunter P. Optical and electro-optical properties of submicrometer lithium niobate slab waveguides prepared by crystal ion slicing and wafer bonding. Appl. Phys. Lett. 85, 4603 (2004).

[b8] Poberaj G., Koechlin M., Sulser F. & Günter P. High-density integrated optics in ion-sliced lithium niobate thin films. In Proc. SPIE, volume 7604, 76040U 1–9 (2010).

[b9] Tamir T. Guided-Wave Optoelectronics, volume 26. Springer (1988).

[b10] Thomas J. *et al.* Laser direct writing: Enabling monolithic and hybrid integrated solutions on the lithium niobate platform. physica status solidi (a) 208(2), 276–283 (2011).

[b11] Ulliac G. *et al.* Ultra-smooth LiNbO3 micro and nano structures for photonic applications. Microelectron. Eng. 88(8), 2417–2419 (2011). Proceedings of the 36th International Conference on Micro- and Nano-Engineering (MNE)36th International Conference on Micro- and Nano-Engineering (MNE).

[b12] Takigawa R., Higurashi E., Kawanishi T. & Asano T. Lithium niobate ridged waveguides with smooth vertical sidewalls fabricated by an ultra-precision cutting method. Opt. Express 22(22), 27733–27738 (2014). 25401917 10.1364/OE.22.027733

[b13] Guarino A., Poberaj G., Rezzonico D., Degl’Innocenti R. & Günter P. Electro–optically tunable microring resonators in lithium niobate. Nat. Photonics 1(7), 407–410 (2007).

[b14] Mookherjea S. & Savanier M., inventors. Heterogeneous waveguide and methods of manufacture. United States provisional patent 62114884. 2015 Feb. 11.

[b15] Han H., Cai L. & Hu H. Optical and structural properties of single-crystal lithium niobate thin film. Opt. Mater. 42, 47–51 (2015).

[b16] Takagi H., Maeda R., Hosoda N. & Suga T. Room-temperature bonding of lithium niobate and silicon wafers by argon-beam surface activation. Appl. Phys. Lett. 74(16), 2387–2389 (1999).

[b17] Howlader M., Suga T. & Kim M. Room temperature bonding of silicon and lithium niobate. Appl. Phys. Lett. 89(3), 031914 (2006).

[b18] Takigawa R., Higurashi E., Suga T., Shinada S. & Kawanishi T. Low-temperature bonding of a LiNbO3 waveguide chip to a Si substrate in ambient air for hybrid-integrated optical devices. Proc. SPIE 6376, 637603–7 (2006).

[b19] Lee Y. S. *et al.* Hybrid Si-LiNbO3 microring electro-optically tunable resonators for active photonic devices. Opt. Lett. 36(7), 1119–1121 (2011). 21479002 10.1364/OL.36.001119

[b20] Chen L., Xu Q., Wood M. G. & Reano R. M. Hybrid silicon and lithium niobate electro-optical ring modulator. Optica 1(2), 112–118 (2014).

[b21] Chiles J. & Fathpour S. Mid-infrared integrated waveguide modulators based on silicon-on-lithium-niobate photonics. Optica 1(5), 350–355 (2014).

[b22] Yoon J. & Kim K. Growth of highly textured LiNbO3 thin film on Si with MgO buffer layer through the solgel process. Appl. Phys. Lett. 68(18), 2523–2525 (1996).

[b23] Sakashita Y. & Segawa H. Preparation and characterization of LiNbO3 thin films produced by chemical vapor deposition. J. Appl. Phys. 77(11), 5995–5999 (1995).

[b24] Chen L. & Reano R. M. Compact electric field sensors based on indirect bonding of lithium niobate to silicon microrings. Opt. Express 20(4), 4032–4038 (2012). 22418161 10.1364/OE.20.004032

[b25] Hu H., Ricken R. & Sohler W. Etching of lithium niobate: from ridge waveguides to photonic crystal structures. *ECIO, Eindhoven*, WeD3, (2008).

[b26] Rabiei P. & Steier W. H. Lithium niobate ridge waveguides and modulators fabricated using smart guide. Appl. Phys. Lett. 86(16), 161115 (2005).

[b27] Almeida V. R., Panepucci R. R. & Lipson M. Nanotaper for compact mode conversion. Opt. Lett. 28(15), 1302–1304 (2003). 12906070 10.1364/ol.28.001302

[b28] Uchida N. Optical waveguide loaded with high refractive-index strip film. Appl. Opt. 15(1), 179–182 (1976). 20155202 10.1364/AO.15.000179

[b29] Hu H., Ricken R. & Sohler W. Lithium niobate photonic wires. Opt. Express 17(26), 24261–24268, Dec (2009). 20052137 10.1364/OE.17.024261

[b30] Vlasov Y. & McNab S. Losses in single-mode silicon-on-insulator strip waveguides and bends. Opt. Express 12(8), 1622–1631 (2004). 19474988 10.1364/opex.12.001622

[b31] Bures J. Guided Optics. WILEY-VCH Verlag, (2009).

[b32] Rao A. *et al.* Heterogeneous microring and mach-zehnder lithium niobate electro-optical modulators on silicon. In *CLEO: Science and Innovations*, STu2F–4. Optical Society of America, (2015).

[b33] Ogusu K. & Tanaka I. Optical strip waveguide: an experiment. Appl. Opt. 19, 3322–3325 (1980). 20234615 10.1364/AO.19.003322

[b34] Cai L., Han S. L. H. & Hu H. Waveguides in single-crystal lithium niobate thin film by proton exchange. Opt. Express 23(2), 1240–1248 (2015). 25835882 10.1364/OE.23.001240

[b35] Li S., Cai L., Wang Y., Jiang Y. & Hu H. Waveguides consisting of single-crystal lithium niobate thin film and oxidized titanium stripe. Opt. Express 23(19), 24212–24219 (2015). 26406627 10.1364/OE.23.024212

[b36] Schmidt R., Cross P. & Glass A. Optically induced crosstalk in linbo3 waveguide switches. J. Appl. Phys. 51(1), 90–93 (1980).

[b37] Mueller C. T. & Garmire E. Photorefractive effect in linbo3 directional couplers. Appl. Opt. 23(23), 4348–4351 (1984). 18213321 10.1364/ao.23.004348

[b38] Turner A. C. *et al.* Tailored anomalous group-velocity dispersion in silicon channel waveguides. Opt. Express 14(10), 4357–4362 (2006). 19516587 10.1364/oe.14.004357

[b39] Aguinaldo R., Shen Y. & Mookherjea S. Large dispersion of silicon directional couplers obtained via wideband microring parametric characterization. IEEE Photon. Technol. Lett. 24(14), 1242–1244 (2012).

[b40] Plach T. *et al.* Mechanisms for room temperature direct wafer bonding. J. Appl. Phys. 113(9), 094905 (2013).

[b41] Vallin Ö., Jonsson K. & Lindberg U. Adhesion quantification methods for wafer bonding. Mat. Sci. Eng. R. 50(4), 109–165 (2005).

