# Supplementary material for: Lightwave Circuits in Lithium Niobate through Hybrid Waveguides with Silicon Photonics
Source: Sci Rep. 2016 Mar 1;6:22301. doi: 10.1038/srep22301 (PMC4772107; doi:10.1038/srep22301)
Supplement: Supplementary Information [file srep22301-s1.pdf]

Lightwave Circuits in Lithium Niobate through Hybrid Waveguides with Silicon Photonics: Supplementary Information

Peter O. Weigel,[1,*] Marc Savanier,[1] Christopher DeRose,[2] Andrew T. Pomerene,[2] Andrew L. Starbuck,[2] Anthony L. Lentine,[2] Vincent Stenger,[3] and Shayan Mookherjea[1,†]

[1]*Department of Electrical & Computer Engineering,*

University of California, San Diego,

La Jolla, California 92093, USA

[2]*Sandia National Laboratory, Applied Microphotonic Systems,*

Albuquerque, New Mexico 87185 USA

[3]*SRICO Inc., 2724 Sawbury Blvd Columbus, OH 43235-4579 USA*

As described in the main text of our paper, two types of Mach-Zehnder Interferometers (MZIs) were designed and measured; the two structures differ in using hybrid waveguides of cross-sections A (Si-like) and B (LN-like), respectively, in the path-length difference section.

Figure S1 shows the 'Cross' and 'Thru' transmissions measured at the two output ports for each input port (a total of four combinations for each MZI structure) while the laser wavelength was varied between 1520 nm and 1620 nm. The sum of these two quantities is also plotted, which is seen to be constant with wavelength, as expected. A narrow wavelength band near 1585 nm has a transmission artifact which is excluded from the data analysis.

We used these datasets to calculate the modal effective indices (n_{eff}) of the cross-sections A and B, and the (length-integrated) coupling coefficient ($|\kappa|^2$) of the directional couplers, and compare the extracted values with a computational model.

We modeled the transfer characteristics ($i = \{A, B\}$) using the following expressions derived by a transfer-matrix formalism:

$$P_{i,thru} = K \left[\left(a\,|t|^2 - |\kappa|^2\right)^2 + 4\,a\,|\kappa|^2\,|t|^2 \sin^2\left(\tfrac{\pi}{\lambda} n_{\text{eff}}\, L_i\right) \right] \quad (1a)$$

$$P_{i,cross} = K\,|\kappa|^2\,|t|^2 \left[(a-1)^2 + 4\,a\cos^2\left(\tfrac{\pi}{\lambda} n_{\text{eff}}\, L_i\right) \right] \quad (1b)$$

where a is the additional field attenuation in the long arm of the MZI (known from the propagation loss measurements), λ is the wavelength and $|t|^2$ is the (length-integrated) transmission coefficient of the directional couplers. We assume that the two directional couplers in each MZI are identical. The prefactor K was estimated from the fit of $P_{thru} + P_{cross} \approx K a^2$ with a constant function. We used a least-square Levenberg-Marquardt (LM) nonlinear fitting algorithm, in which the 'unknown' parameters n_{eff} and $|\kappa|^2$ were parameterized with a second-order and a first-order polynomial expansion, respectively, in the wavelength λ. As a starting point for the LM algorithm, we initialized the variables using values estimated from numerical simulations. The results of each independent fitting procedure are displayed in Fig. S1 using solid lines, and which show very good agreement between the model and the experimental data.

The key parameters in the model affect the measured transmission response in a few different ways. The variation of n_{eff} with wavelength is reflected in the change of the spectral periodicity of the transfer function with wavelength. The variation of $|\kappa|^2$ with wavelength is reflected in the visibility of the two-wave interference pattern and the wavelength-dependent imbalance between the two arms of the MZI. The observation that the fringes diminish in

contrast as λ approaches 1620 nm even though $P_{\text{thru}} + P_{\text{cross}}$ remains constant suggests that the magnitude of the coupling coefficient approaches 1 at the longer wavelengths. Physically, when $|\kappa|^2 = 1$, all the light is transferred from one arm to the other, so that no interference occurs and the 'Thru' port is always 'on'. Altering the lithographic photomask to vary the Si rib width will result in shifting the wavelength at which this behavior occurs to different values. Although we were unable to achieve a 50-50 coupling ratio at 1550 nm, such an error can be corrected using a mask bias in future lithography iterations.

For each device, the same fitting procedure was independently performed on the transfer characteristics ($\text{in}_{\{1,2\}} \rightarrow \text{out}_{\{1,2\}}$). From the four sets of extracted coefficients, we report the average $n_{\text{eff}}(\lambda)$ for both cross-sections A and B in Fig. 5b (main text). In Fig. 5c (main text), we plot the directional coupler power-coupling coefficient $|\kappa|^2(\lambda)$ as a function of wavelength.

The standard errors provided by the fitting routine (performed in a numerical data analysis software package) were propagated using the algebraic equations which defined n_{eff} and $|\kappa|^2$ as functions of wavelength to estimate the uncertainty on the ensemble-averaged extracted values of both n_{eff} and $|\kappa|^2$, and are shown by the shaded regions in Figs. 5b (main text) and 5c (main text).

These observations explain the notable difference in extinction ratio (ER) for the 'Thru' and the 'Cross' transmissions, which is a symptom of non-3-dB directional couplers ($|\kappa|^2 \neq 0.5$). (As an aside, the fact that $|\kappa|^2 > 0.5$ between 1520 nm and 1620 nm, as displayed in Fig. 5c, confirms that $\text{ER}_{\text{thru}} < \text{ER}_{\text{cross}}$ over the wavelength range in Fig. S1.) Armed with this knowledge, we anticipate being able to correct such deterministic errors by slightly changing the lithographic mask in future fabrication iterations.

The flatness of the $\text{out}_1 + \text{out}_2$ traces in all the cases shows that the coupled-mode model of the directional coupler provides a satisfactory fit over a wavelength range of about 100 nm, which is sufficient for wavelength-division multiplexed optical devices in the C-band telecommunications wavelengths. For nonlinear optical devices, e.g., wavelength converters, the couplers may have to be studied in more detail at multiple spectral bands.

---

* Electronic address: pweigel@eng.ucsd.edu

† Electronic address: smookherjea@ucsd.edu

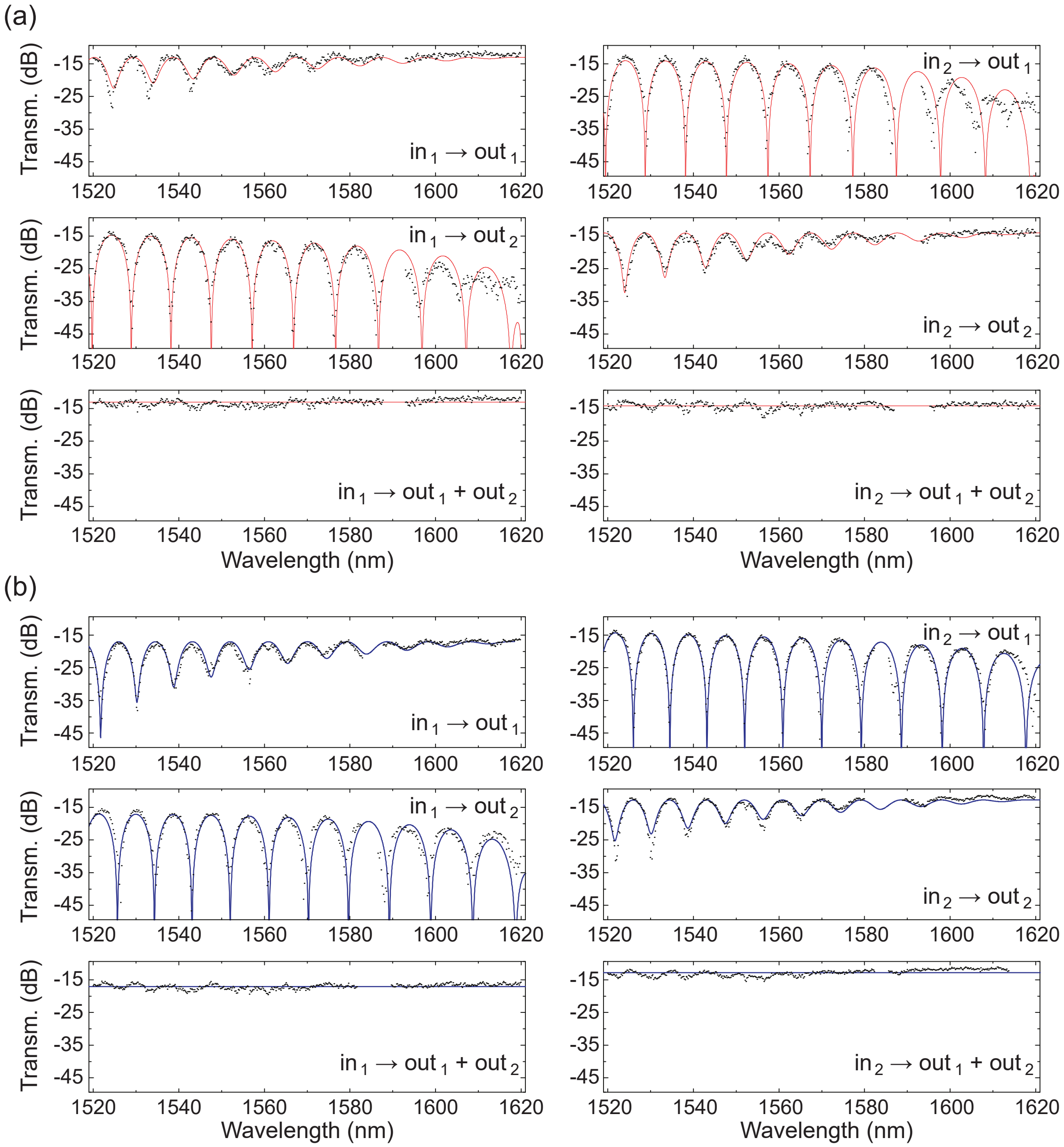

FIG. S1: Two types of Mach-Zehnder interferometers (MZIs) were fabricated, following the general layout shown in Fig. 5 (main text) and using hybrid waveguides with cross-sections A and B, respectively, in the path-length difference section. The input-to-output optical transmission was measured (four combinations in each case, from the two input and two output ports of the MZI). Black dots report the experimental measurements and solid lines show the fitted response using a transfer-matrix model, as described in this Supplementary Information text.
